# Probiotics supplementation or probiotic-fortified products on sarcopenic indices in older adults: systematic review and meta-analysis from recent randomized controlled trials

**DOI:** 10.3389/fragi.2024.1307762

**Published:** 2024-02-02

**Authors:** Yvonne Suzy Handajani, Yuda Turana, Antoninus Hengky, Gabriella Hamid, Elisabeth Schroeder-Butterfill, Kevin Kristian

**Affiliations:** ^1^ School of Medicine and Health Sciences, Atma Jaya Catholic University of Indonesia, Jakarta, Indonesia; ^2^ Center of Health Research, Atma Jaya Catholic University of Indonesia, Jakarta, Indonesia; ^3^ Fatima General Hospital, Ketapang Regency, West Kalimantan, Ketapang, Indonesia; ^4^ Leona Kefamenanu General Hospital, North Central Timor, Indonesia; ^5^ Department of Gerontology, University of Southampton, Southampton, NH, United Kingdom

**Keywords:** probiotic, older adult, sarcopenia, muscle mass, muscle strength

## Abstract

**Introduction:** A noteworthy correlation was seen between changes in the gut microbiome and sarcopenia in older adults. Along with increasing research on probiotic supplementation for various medical problems, we aimed to obtain evidence and summarize the effect of probiotic supplementation on sarcopenic indices among older adults.

**Methods:** We utilized PubMed, EBSCO, and Proquest, in addition to manual search using synonyms and variation for ‘probiotic,’ ‘sarcopenia,’ and ‘older adults.’ Randomized controlled trials investigated the utilization of probiotics or probiotic-containing products in older adults with sarcopenic indices including muscle mass and strength. The random-effects model was applied to the meta-analysis process.

**Results:** Seven studies were obtained with 733 pooled older adults. Probiotic supplementation resulted in a significant increase of muscle mass with adjusted SMD (Standardized Mean Difference) of 0.962 (95% CI: 0.288 to 1.635, *p* = 0.049) using till and trim analysis and muscle strength with SMD of 1.037 (95% CI: 0.077 to 1.996, *p* = 0.03). However, both outcomes were associated with significantly high heterogeneity (I^2^ = 89.5% and I^2^ = 89.9%, respectively).

**Conclusion:** When opposed to a placebo, the probiotic treatment improved the amount of muscle and its endurance based on recent evidence, however, further studies should be done with larger samples and targeted populations.

## Introduction

There’s a shift in Indonesia’s population toward older ages. One in six persons in the world will be 60 years old or more by 2030, based on World Health Organization data. World Health Organization [WHO], (2023) Additionally, the average life expectancy at birth has risen significantly, rising from 47 years in 1950 to 72 years in 2020. World Health Organization [WHO], (2019). This population aging comes with health consequences. Numerous age-related diseases, such as cancer, immune system disorders, musculoskeletal disorders, and neurological diseases, are driven by aging. Li et al. (2021) Of particular note within the domain of musculoskeletal disorders associated with aging are sarcopenia and osteoarthritis (OA). Grote et al. (2019).

Muscular mass and strength drop over time as people get older, but sarcopenic people have been shown to experience an accelerated decline in muscular function. Grote et al. (2019) Sarcopenia is age-related loss of skeletal muscle mass plus loss of muscle strength and/or reduced physical performance. Bahat et al. (2016); Chen et al. (2020) Meta-analyses conducted by Peterman-Rocha et al. have indicated a prevalence range of sarcopenia spanning from 10% to 27% among individuals aged 60 years or older. Petermann-Rocha et al. (2022) Sarcopenia’s muscle wasting can be an inflammation-driven condition. In normal conditions, environmental stressors like physical activity and protein consumption have an impact on the harmony between the production and degradation of proteins.

The use of probiotic supplements for older adults with a variety of medical illnesses is still being researched, with neuropsychiatric conditions like dementia, mood disorders, and autism spectrum disorder making up the majority of the conditions. Sandhu et al. (2017); Rudzki et al. (2019); Handajani et al. (2020); Handajani et al. (2023) All of them were predicated on the theory that the microbiome-gut-brain axis could influence brain physiology. Additionally, the host’s physiology, endocrinology, and immune system are significantly influenced by the gut bacteria. Sharon et al. (2016); Sandhu et al. (2017) The age of the host has an impact on the variety of the microbiota, and these changes may have an impact on how quickly people age and develop diseases. Salazar et al. (2014). In a study by Liu et al., sarcopenia in older persons was significantly correlated with the diversity of specific bacteria. Liu et al. (2021) Furthermore, it is hypothesized that sarcopenia’s muscle wasting may be driven, in part, by inflammation. Under ordinary circumstances, the ratio of protein production to breakdown is adjusted in response to external stimuli like exercise and protein from the diet intake playing essential roles. An intriguing hypothesis posits that probiotics may mitigate muscle wasting by potentially reducing gut permeability. Such modulation is believed to contribute to muscle deterioration associated with sarcopenia van Krimpen et al. (2021).

The article aimed to systematically explore the utilization of probiotic supplementation effects on sarcopenic indices in older persons.

## Methods

This systematic review was performed based on Preferred Reporting Items for Systematic Review and Meta-Analysis (PRISMA) guidelines (CRD42023466881).

### Inclusion criteria

Randomized controlled trials (RCTs) that investigate the utilization of probiotics or probiotic-containing products in older adults with sarcopenic indices including muscle mass and strength as outcome measures. Studies other than RCTs were excluded from this review. Comparators in these studies could be placebo or standard treatment.

### Search methodology

We utilized three databases, including PubMed, EBSCO, and Proquest, in addition to a manual search of reference lists of relevant research or reviews. The search covered the synonyms and variations for ‘probiotic,’ ‘sarcopenia,’ and ‘older adults’ using medical subject headings (MeSH) and free text terms (Supplementary File S1) with English or Indonesian language and no year of publication restriction.

### Data selection, collection, and extraction

We used Zotero as our reference manager. Following the collection of the identified studies, duplicates were eliminated, and titles and abstracts were used to assess the studies for eligibility. Two co-authors (AH and GH) worked independently on this process. After the initial screening, all studies that might be relevant will go through a full-text evaluation independently. If there are any disagreements during the selection and quality evaluation processes, the remaining co-authors (YSH and YT) will discuss them. Following full-text evaluation, studies that met the requirements were extracted for synthesis and summary of the results by working together (AH and GH). Data were confirmed by other co-authors (YSH and YT). Author, year, country, population characteristics included in the study, allocations, total samples, intervention, and comparator (types and quantities of probiotic), duration of study, outcome measure, outcomes at the end of study (muscle mass and strength), and general findings were all collected from the study.

### Quality assessment

We used RoB2 to assess the quality of RCT studies that we included.

### Data analysis and synthesis

We did both qualitative and quantitative synthesis. In qualitative synthesis, we present the summary of included studies, elaboration of these studies, and discuss the possible benefits of probiotic supplementation, in addition to providing an elaboration of possible mechanisms based on existing evidence. Probiotics’ impact on lean muscle mass and strength was measured in a meta-analysis using a model with random effect. I^2^ was used to present heterogeneity and treat it qualitatively. In order to assess publication bias, Egger’s test was applied, and then fill and trim analysis was used to correct it. Relevant data will be combined and calculated using statistical software Comprehensive Meta-Analysis version 3.

## Results

### Study characteristics

**FIGURE 1:**
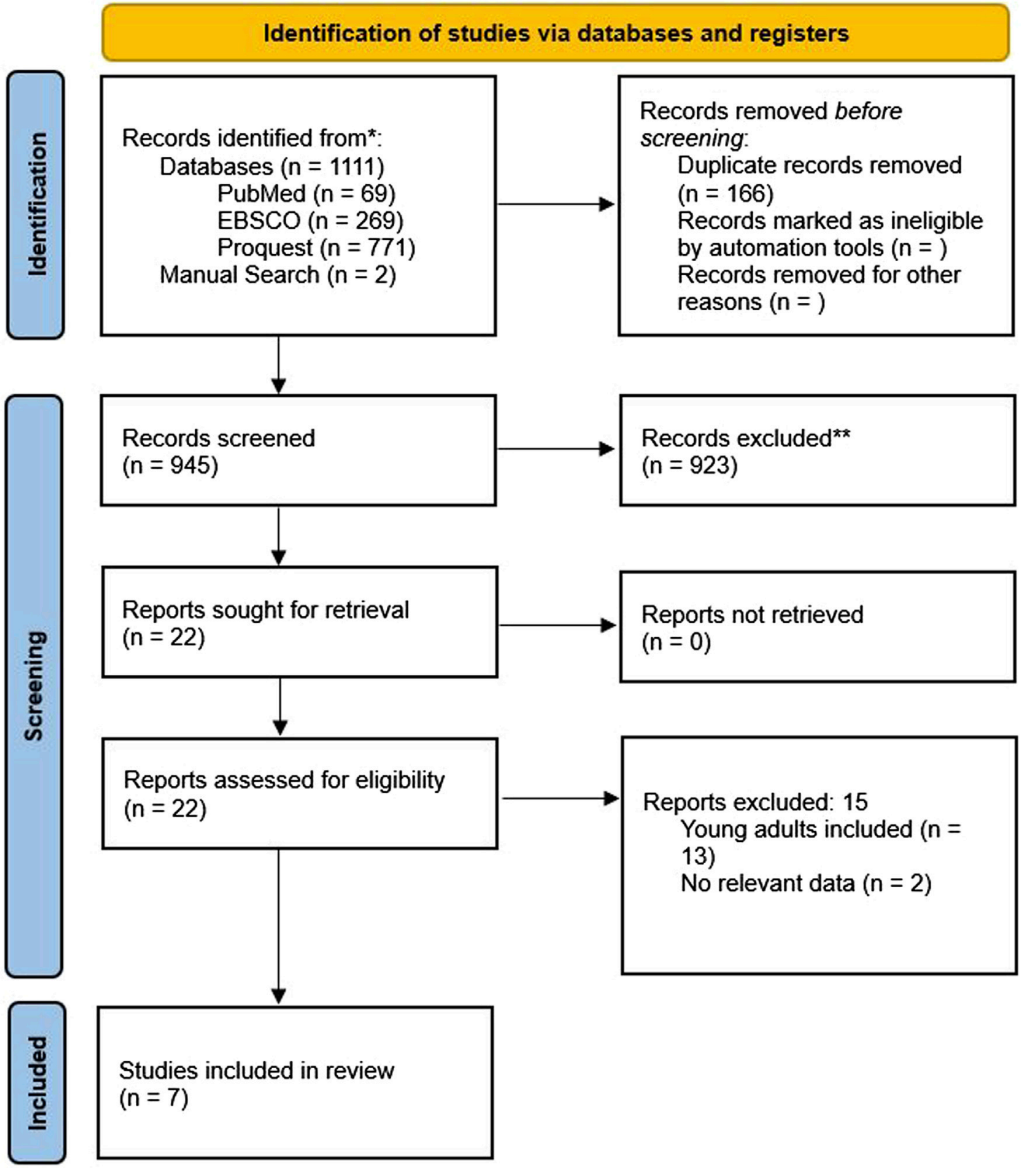
PRISMA flow diagram.

**FIGURE 2:**
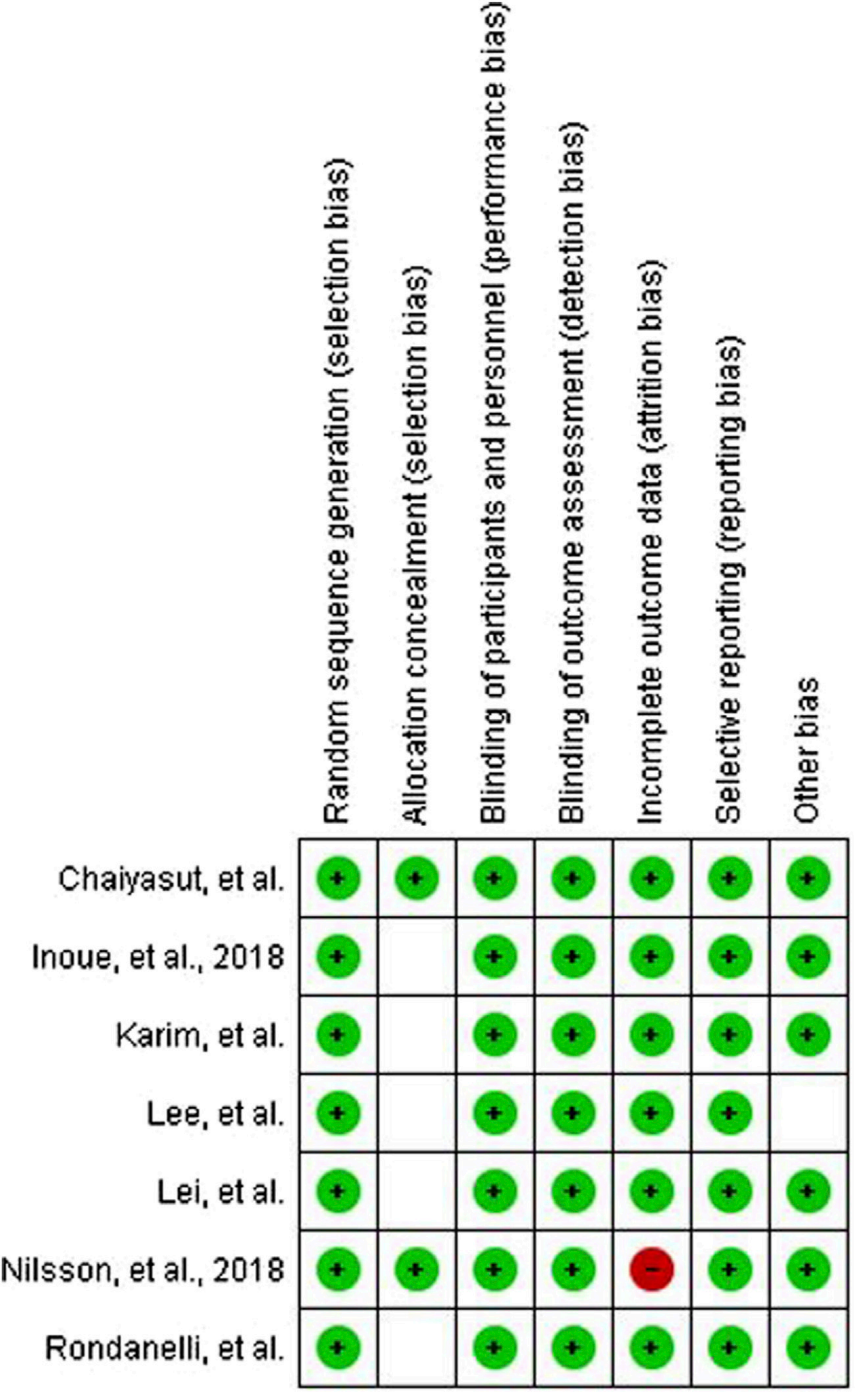
Risk of bias assessment using RoB2.

We initially found 1,111 studies by searching through databases and conducting manual searches (Figure 1). After removing 166 duplicates, we screened 945 studies, leading to 22 studies for a thorough examination of their full text. Ultimately, we decided to include 7 studies in this review. Some studies were excluded due to the absence of pertinent data and because they involved younger populations. Figure 2 displays the evaluation of the risks of bias.

In these 7 studies, there were a total of 733 pooled participants, with quite heterogenous populations (Table 1). Three studies used a mixture of multistrain probiotics, while the other 4 used single-strain probiotics with follow-up ranging from 8 weeks to 12 months.

**TABLE 1:** Characteristics of included studies.

Author, year	Country	Included population	Allocation		Intervention	Control	Outcome measure	Duration of study	General findings
			Intervention	Control					
Chaiyasut, et al. (2022)	Thailand	Healthy older adults	24	24	Mixture of probiotics (2.0 × 10^10 CFU of *L. paracasei* HII01; 2.0 × 10^10 CFU of *B. breve*; 1.0 × 10^10 CFU of *B. longum*) (Lactomason Co., Ltd., Jinju-si, South Korea)	10 g of corn starch in similar package of probiotics	Muscle Mass (%)	12 weeks	The intervention improved obesity-related anthropometric biomarkers (body fat %, visceral fat %, muscle %, arm, waist, and hip circumference), short-chain fatty acids, and intestinal barrier function. HDL-C was increased in intervention groups. Adverse event was not reported
Karim et al. (2022)	UAE	Older adults with CHF	48	44	Mixture of probiotics (*B. longum* DSM 24736, *B. breve* DSM 24732, DSM 24737, lactobacilli DSM 24735, DSM 24730, DSM 24733, *L. delbrueckii* subsp. bulgaricus DSM 24734, and *S. thermophilus* DSM 24731) (11.2 × 10^10^ CFU) (Vivomixx^®^ 112)	Inactive agent	ASM (kg) HGS (kg)	12 weeks	Probiotics intervention improved HGS, gait speed, and plasma Dkk-1. No significant difference in ASM, fat mass%, BMI, and ASMI was observed. Adverse event was not reported
Lee et al. (2021)	Taiwan	Older adults with frailty	36	19	*L. plantarum* isolated from Taiwanese pickled cabbage cultivated (TWK10) (Synbio Tech Inc., Kaohsiung, Taiwan). TWK-10-H (high-dose group) = 6 × 10^10^ CFU/day TWK10-L (low-dose group) = 2 × 10^10^ CFU/day	The composition was similar to TWK10 capsules, but TWK10 was not added	Muscle Mass (kg) HGS (kg)	18 weeks	Intervention increase muscle mass, HGS, lower limb muscle strength, gait speed, and balance, especially in TWK10-H group. Adverse event was not reported
Inoue et al. (2018)	Japan	Healthy older adults	20	19	Mixture of *B. longum* BB536, *B. infantis* M-63, *B. breve* M-16 V, and *B. breve* B-3 (1.25 × 10^10^ CFU each) (Morinaga Milk Industry Co., Ltd., Kanagawa, Japan) with resistance training programme	Placebo (dextrin with water) with resistance training programme	LBM (kg)	12 weeks	There were significant decrease of body composition (body mass, BMI, and fat %) within probiotic group, however, changes in body composition between the groups were not significant. There was no adverse events among participants
Nilsson et al. (2018a)	Sweden	Post menopausal older adults	32	36	*L. reuteri* 6,475 (1 × 10^10 CFU) with maltodextrin (BioGaia AB, Stockholm, Sweden)	Maltodextrin powder	LBM (kg)	12 months	There was no significant difference in total lean mass between groups. Adverse events were reported in 80% and 87% participants of probiotic and placebo groups, respectively. The most common adverse events was gastrointestinal disorder such as change of bowel habit, flatulence, and nausea and vomiting
Rondanelli et al. (2022)	Italy	Older adults with sarcopenia	22	28	Formulated product: probiotic *L. paracasei* PS23 (3 × 10^10^ CFU), omega-3 fatty acid 500 mg, leucine 2.5 g, and combined with physical activity and dieatary program	Isocaloric placebo with the same flavor with physical activity and dieatary program	ALM (g) HGS (kg)	8 weeks	ALM and HGS improved and visceral fat decreased significantly in the intervention group compared to placebo. Body weight, BMI, and waist circumference increased in intervention group. There was no intervention-related adverse events among participants
Lei et al. (2016)	China	Older adults with radius fracture	189	192	Skimmed milk containing a minimum of 6 × 109 cfu *L. casei* Shirota twice daily	Skimmed milk	HGS (kg)	6 months	There significant improvement in HGS among intervention group in early phase (2–5 months) of the study, however, no difference was observed in the end (6 months) of the study. Adverse event was not reported

CFU, colony forming unit; CHF, chronic heart failure; ASM, appendicular skeletal mass; HGS, handgrip strength; LBM, lean body mass; ALM, appendicular lean mass.

### Probiotics on muscle mass

**FIGURE 3:**
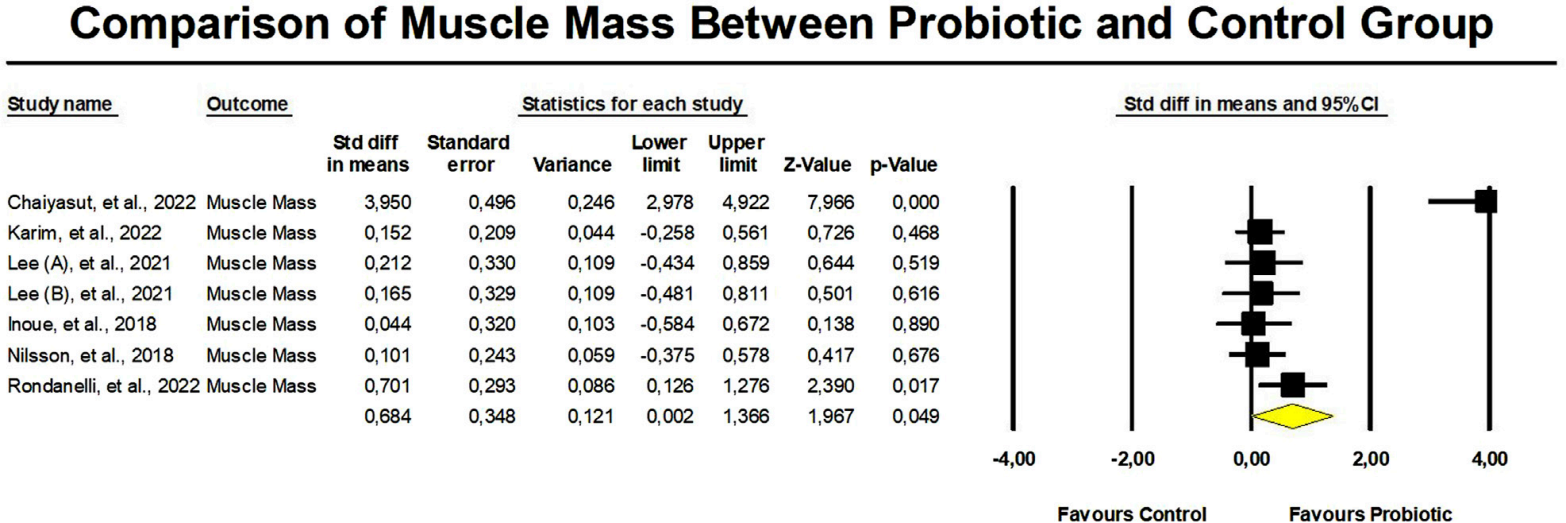
Meta-analysis of probiotic supplementation in muscle mass.

**FIGURE 4:**
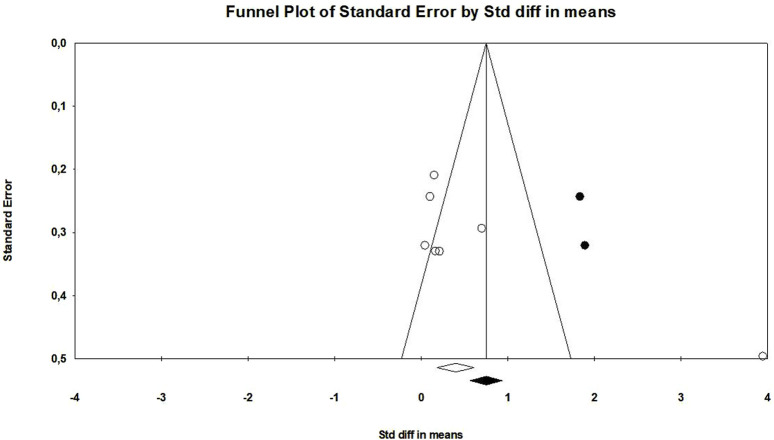
Funnel plot of muscle mass outcome.

The probiotic group displayed a significant increase in muscle mass in contrast to the control group, with a standardized mean difference (SMD) of 0.684 (95% confidence interval: 0.002 to 1.366, *p* = 0.049) (Figure 3). However, there was a significant heterogeneity (I2 = 89.5%, *p* < 0.01) in these results. Additionally, there was proof of a major bias in publications (Egger’s *p* = 0.03) in this outcome. To address this, we performed a trim-and-fill analysis, which provided an adjusted SMD value of 0.962 (95% CI: 0.288–1.635) (Figure 4).

### Probiotics on muscle strength

**FIGURE 5:**
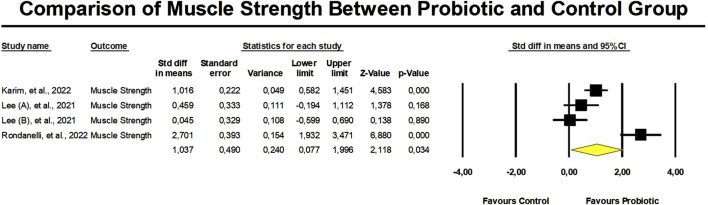
Meta-analysis of probiotic supplementation in muscle strength.

There was a significant improvement in muscle strength in the probiotic group in contrast to the control group, as mentioned by an SDM of 1.037 (95% confidence interval: 0.077 to 1.996, *p* = 0.03) (Figure 5). Nevertheless, there was a significantly high heterogeneity in these findings (I2 = 89.9%, *p* < 0.01). It is crucial to remember that there was no proof of publication bias (Egger’s *p* = 0.38).

## Discussions

Recent research suggested that probiotic supplementation may have some benefits for muscle strength and mass, particularly for old persons. However, these studies pose some concerns, primarily due to the differences in the included population. Although only older adults were included, some studies used older adults with already existing sarcopenia or frailty, the others used patients with CHF (chronic heart failure), postmenopausal women, and patients with fractures. Lei et al. (2016); Nilsson et al. (2018a); Lee et al. (2021); Karim et al. (2022); Rondanelli et al. (2022) The effects of probiotics on each of these conditions were unknown to the overall muscle mass and strength. Most of these studies had positive outcomes, although some were not significant. In our study, three out of six studies that measure muscle mass used bioelectrical impedance analysis, while the other three used dual-energy x-ray absorptiometry, however, the brands were different and the technologies may vary. Four studies that measure muscle strength, all used a hand dynamometer to measure handgrip strength. Lean body mass or muscle mass can be measured by different modalities, such as dual energy x-ray absorptiometry, computed tomography, magnetic resonance imaging, and bioelectrical impedance analysis. Tosato et al. (2017); Buckinx et al. (2018) There are no currently available techniques that serve all requirements for muscle mass measurement, each has its advantages and disadvantages. Buckinx et al. (2018) The results between measurements might differ and affect the overall results. This also applies to muscle strength which can be measured using manual muscle testing, field tests, hand-held dynamometry, and handgrip dynamometry. However, according to AWGSOP/EWGSOP, for standardized measurement, muscle mass should be measured using dual-energy x-ray absorptiometry or bioelectrical impedance analysis while muscle strength uses handgrip strength Bahat et al. (2016); Chen et al. (2020).

Chaiyasut et al. also found improved intestinal barrier function by up to 48%, obesity-related anthropometric biomarkers (hip and waist circumference, muscle, body fat, and BMI), and short-chain fatty acids after supplementation with multistrain probiotics containing *L. paracasei* HII01, *B. breve*, *B. longum* in healthy elderly people Chaiyasut et al. (2022). Levels of HDL-C increased significantly, but no significant changes to TC, hsCRP, LDL-C, and TG were observed. Lee et al. used *L. plantarum* TWK10 and found significant improvement in muscle strength and mass, balance, and walking speed in older adults with frailty, especially in the high-dose group with 6 × 10^10^ CFU/day Lee et al. (2021). No significant improvement in bone mineral density was observed. Probiotic supplementation was added to the training program of healthy elderly people with normal BMI in Inuoe et al.’s study, which led to a significant decrease in body weight, BMI, and fat percentage, but no difference in lean body mass Inoue et al. (2018). Some studies have found that supplementation of probiotics might have body fat-decreasing impacts. Kondo et al. showed that B. breve B-3 prevented mice fed a diet high in fat from gaining weight or accumulating fat Kondo et al. (2010). This probiotic might also affect the regulation of gene expression responsible for lipid metabolism Kondo et al. (2013). The *L. reuteri* 6,475 supplementations in postmenopausal women significantly reduced bone loss in Nilsson et al., however, there was no difference in total lean mass or inflammatory markers (N-terminal telopeptide, ALP, CRP, and TNF-α) between both groups. Nilsson et al. (2018a) In in vivo studies, *L. reuteri* 6,475 could interfere with TNF-α-mediated inflammation, however, it did not show significant changes in body compositions. Komaroff, (2017); Nilsson et al. (2018b) Rondanelli et al. is the only study that investigated the effects of probiotic *L. paracasei* PS23 for sarcopenia in older adults. Lean mass of the appendices and strength of grip was significantly increased in the group that consumed probiotics compared to placebo, with decreased visceral adiposity. Rondanelli et al. (2022) Amino acids including valine, leucine, and isoleucine, and all combined also increased significantly. Administration of probiotics (*L. paracasei* LPC-S01 and *L. paracasei* LP-DG) might also improve amino acids absorption, therefore probiotic supplementation should be combined with amino acids. Jäger et al. (2020) Several studies have demonstrated that leucine might help alter muscular protein turnover by enhancing production and reducing proteolysis. Balage and Dardevet, (2010); Dai et al. (2021) Intake of 3 g leucine combined with 25–30 protein was found to be beneficial to prevent loss of lean mass in older adults. Kim et al. (2012) Lei et al. study that investigated probiotics on older adults with distal radius fracture found the acceleration of the healing process, leading to faster handgrip strength recovery compared to placebo. Muscle strength improved faster in the early 1–5 months, however, both groups ended up with no difference in strength after 6 months of follow-up. Lei et al. (2016) Mutistrain probiotics investigated by Karim et al. in CHF patients also showed favorable effects of probiotics on handgrip strength, gait speed, and Wnt family protein (Dkk-1, Dkk-3, and SREBP1). Karim et al. (2022) Wnt signaling plays an essential role in myogenesis, muscle repair, and stem cell regeneration. Arthur and Cooley, (2012).

**FIGURE 6:**
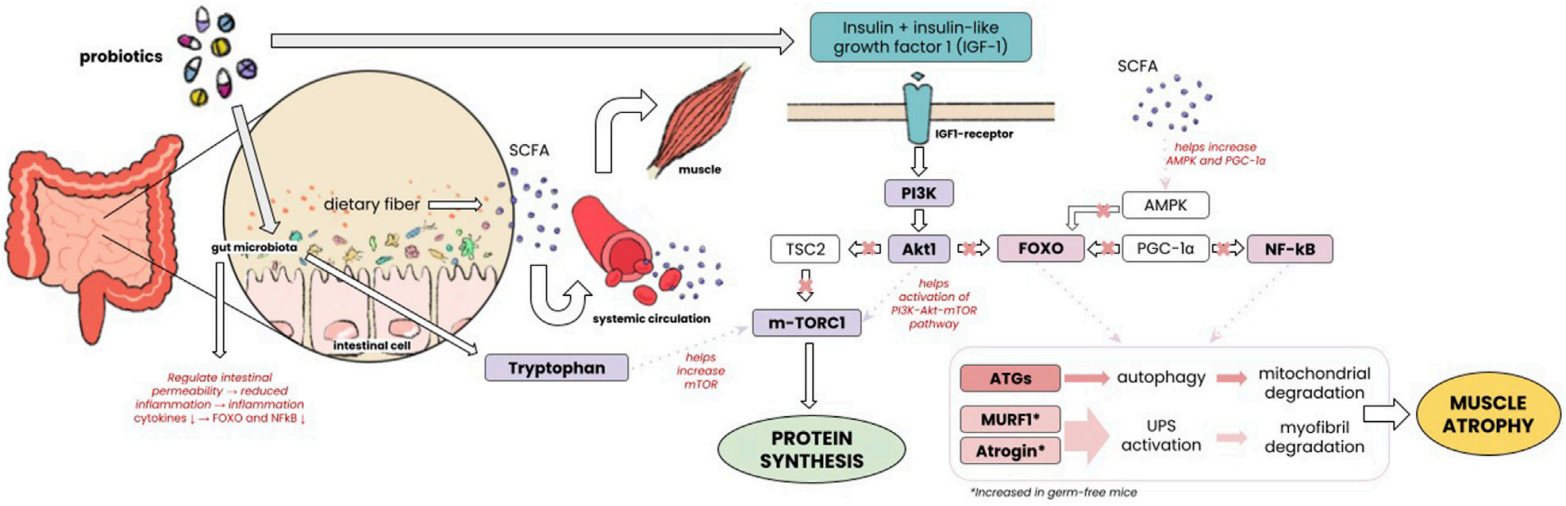
Mechanism of gut microbiome affecting muscle mass.

In this study, probiotic administration led to a considerable increase in muscle mass. Multiple animal studies have shown that supplementation with probiotics affects muscle mass by enhancing muscle weight, muscle fiber size, and the size of the tibialis muscle. Bindels et al. (2012); Sugimura et al. (2022) Some studies have tried to explain the mechanism of this finding. Muscle mass is maintained by the balance between its synthesis and degradation. Muscle synthesis involving insulin-like growth factor (IGF-1), Akt/Protein Kinase B-mTOR pathway promotes ribosomal biogenesis and translation to form new myofibril protein. Barclay et al. (2019) Insulin resistance and diminished response to the mTOR pathway can be found in sarcopenia. The gut microbiome in this case was linked to increased expression of IGF-1 by increasing the production of tryptophan (Trp). It was also bound to upregulate mTOR/eif4/p70s6k pathway molecules in muscle samples. Dukes et al. (2015) Another study by Lahiri, et al. comparing germ-free mice with mice that had gut microbiota found that germ-free mice had reduced expression of IGF. Lahiri et al. (2019); Nay et al. (2019) *In vivo* and *in vitro* settings have also demonstrated that short-chain fatty acids (SCFA) produced by gut microbiota affect the preservation of skeletal muscle mass, increasing GLUT4 expression, insulin sensitivity, and blood circulation. Haran et al. (2012); Frampton et al. (2020); de Marco Castro et al. (2021) Gut microbiota was found to counteract anabolic resistance by reducing low-grade inflammation from the intestines and improving food digestion that contains proteins, thus favoring muscle protein synthesis. Haran et al. (2012); de Marco Castro et al. (2021) Muscle atrophy involves several pathways such as the ubiquitin-proteasome system (UPS) which involves Atrogin-1/MAFbx and MuRF1. Sartori et al. (2021) It was found that germ-free mice had increased expression of *Atrogin-1* and *Murf-1* which encode E3 ubiquitin ligase involved in muscle atrophy. Lahiri et al. (2019) Muscle catabolism also includes another system such as the autophagy-lysosome system and FoxOs-astrogenes. Sartori et al. (2021) SCFA which is produced by gut microbiome increases the production of AMPK and PGC1α which regulates this FoxO activity. Frampton et al. (2020) Inflammation increases muscle atrophy. SCFA was also demonstrated to affect the breakdown of fat, carbohydrates, and protein in skeletal muscle tissue, maintaining oxidative phenotype, and thus was shown to prevent muscle atrophy and increase muscle strength. Frampton et al. (2020) Detailed pathways of molecular mechanisms in which gut microbiome affects muscle mass can be seen in Figure 6.

Our study brought to light the substantial impact of probiotic supplementation on the improvement of muscle strength. This was in concordance with previous studies in which probiotics were found to increase muscle strength. Buigues et al. (2016) Animal study also revealed similar results, it increased grip strength in rats compared with age-matched controls. Ni et al. (2019) Several studies reviewed by Lustgarten et al. showed an increase in muscle strength and endurance exercise capacity compared to germ-free control. Lustgarten, (2019) *Prevotellaceae, Prevotella, Barnesiella,* and *Barnesiella intestinihominis* were found to be conjunct with higher muscle strength. *Barnesiella* and *Prevotellaceae*, specifically, were learned to contain genes that produce acetate, propionate, and butyrate. These SCFAs were able to increase Nuclear factor erythroid 2-related factor 2 (Nrf2), a regulator of cellular antioxidant defenses, and also bound G-protein coupled receptor (GPR) 41 or GPR43 intracellularly, which in turn activated several pathways including the release of intracellular Ca^2+^; ERK1/2; and inhibition of cAMP accumulation, thus had an effect in increase of skeletal muscle function. Otten et al. (2023) Different ratios of SCFAs influenced higher glucose uptake in C2C12 myotubes, which increased skeletal muscle glucose uptake. Otten et al. (2023) Germ-free mice were also found to have reduced genes encoding Rapsyn and Lrp4 which are essential for neuromuscular junction assembly. Lahiri et al. (2019).

The strength of our study lies in its depth and thorough exploration of the probiotics utilization that may influence sarcopenia risk/status. This demonstrates a comprehensive understanding of this novel topic. This review is the first to evaluate the utilization of probiotics on sarcopenic indices in older adults. We also highlighted the issues related to the heterogeneity of study populations in the literature showing a keen awareness of potential biases. This critical evaluation demonstrates a thoughtful consideration of the available evidence. While we have rightly identified the heterogeneity in study populations as a point of consideration, it also serves as a limitation. The heterogeneity may introduce confounding variables, making it challenging to draw generalizable conclusions from the combined findings. This limitation should be acknowledged, especially if extrapolating findings to specific demographic groups or clinical conditions.

## Conclusion

Probiotic supplementation in older adults may provide benefits on sarcopenic indices, such as muscle mass and strength. Considering the potential benefits and the no serious adverse events reported, probiotics could be suggested especially for older adults. However, further studies should be done with larger samples and targeted populations that might benefit from intervention.

## Data Availability

The original contributions presented in the study are included in the article/Supplementary Materials, further inquiries can be directed to the corresponding author.

## References

[B1] Arthur S. T. Cooley I. D. (2012). The effect of physiological stimuli on sarcopenia; impact of notch and wnt signaling on impaired aged skeletal muscle repair. Int. J. Biol. Sci. 8 (5), 731–760. 10.7150/ijbs.4262 22701343 PMC3371570

[B2] Bahat G. Tufan A. Tufan F. Kilic C. Akpinar T. S. Kose M. (2016). Cut-off points to identify sarcopenia according to European working group on sarcopenia in older people (EWGSOP) definition. Clin. Nutr. 35 (6), 1557–1563. 10.1016/j.clnu.2016.02.002 26922142

[B3] Balage M. Dardevet D. (2010). Long-term effects of leucine supplementation on body composition. Curr. Opin. Clin. Nutr. Metab. Care 13 (3), 265–270. 10.1097/MCO.0b013e328336f6b8 20110810

[B4] Barclay R. D. Burd N. A. Tyler C. Tillin N. A. Mackenzie R. W. (2019). The role of the IGF-1 signaling cascade in muscle protein synthesis and anabolic resistance in aging skeletal muscle. Front. Nutr. 6, 146. 10.3389/fnut.2019.00146 31552262 PMC6746962

[B5] Bindels L. B. Beck R. Schakman O. Martin J. C. Backer F. D. Sohet F. M. (2012). Restoring specific lactobacilli levels decreases inflammation and muscle atrophy markers in an acute leukemia mouse model. PLOS ONE 7 (6), e37971. 10.1371/journal.pone.0037971 22761662 PMC3384645

[B6] Buckinx F. Landi F. Cesari M. Fielding R. A. Visser M. Engelke K. (2018). Pitfalls in the measurement of muscle mass: a need for a reference standard. J. Cachexia Sarcopenia Muscle 9 (2), 269–278. 10.1002/jcsm.12268 29349935 PMC5879987

[B7] Buigues C. Fernández-Garrido J. Pruimboom L. Hoogland A. J. Navarro-Martínez R. Martínez-Martínez M. (2016). Effect of a prebiotic formulation on frailty syndrome: a randomized, double-blind clinical trial. Int. J. Mol. Sci. 17 (6), 932. 10.3390/ijms17060932 27314331 PMC4926465

[B8] Chaiyasut C. Sivamaruthi B. S. Lailerd N. Sirilun S. Khongtan S. Fukngoen P. (2022). Probiotics supplementation improves intestinal permeability, obesity index and metabolic biomarkers in elderly Thai subjects: a randomized controlled trial. Foods 11 (3), 268. 10.3390/foods11030268 35159419 PMC8834517

[B9] Chen L. K. Woo J. Assantachai P. Auyeung T. W. Chou M. Y. Iijima K. (2020). Asian working group for sarcopenia: 2019 consensus update on sarcopenia diagnosis and treatment. J. Am. Med. Dir. Assoc. 21 (3), 300–307. 10.1016/j.jamda.2019.12.012 32033882

[B10] Dai M. Lin T. Yue J. Dai L. (2021). Signatures and clinical significance of amino acid flux in sarcopenia: a systematic review and meta-analysis. Front. Endocrinol. 12, 725518. 10.3389/fendo.2021.725518 PMC8473793 34589057

[B11] de Marco Castro E. Murphy C. H. Roche H. M. (2021). Targeting the gut microbiota to improve dietary protein efficacy to mitigate sarcopenia. Front. Nutr. 8, 656730. 10.3389/fnut.2021.656730 34235167 PMC8256992

[B12] Dukes A. Davis C. El Refaey M. Upadhyay S. Mork S. Arounleut P. (2015). The aromatic amino acid tryptophan stimulates skeletal muscle IGF1/p70s6k/mTor signaling *in vivo* and the expression of myogenic genes *in vitro* . Nutr Burbank Los Angel Cty Calif 31 (7–8), 1018–1024. 10.1016/j.nut.2015.02.011 PMC4465076 26059377

[B13] Frampton J. Murphy K. G. Frost G. Chambers E. S. (2020). Short-chain fatty acids as potential regulators of skeletal muscle metabolism and function. Nat. Metab. 2 (9), 840–848. 10.1038/s42255-020-0188-7 32694821

[B14] Grote C. Reinhardt D. Zhang M. Wang J. (2019). Regulatory mechanisms and clinical manifestations of musculoskeletal aging. J. Orthop. Res. 37 (7), 1475–1488. 10.1002/jor.24292 30919498 PMC9202363

[B15] Handajani Y. Turana Y. Yogiara Y. Widjaja N. Sani T. Christianto G. (2020). Tempeh consumption and cognitive improvement in mild cognitive impairment. Dement. Geriatr. Cogn. Disord. 49, 497–502. 10.1159/000510563 33080604

[B16] Handajani Y. S. Hengky A. Schröder-Butterfill E. Hogervorst E. Turana Y. (2023). Probiotic supplementation improved cognitive function in cognitively impaired and healthy older adults: a systematic review of recent trials. Neurol. Sci. 44 (4), 1163–1169. 10.1007/s10072-022-06540-8 36529793

[B17] Haran P. H. Rivas D. A. Fielding R. A. (2012). Role and potential mechanisms of anabolic resistance in sarcopenia. J. Cachexia Sarcopenia Muscle 3 (3), 157–162. 10.1007/s13539-012-0068-4 22589021 PMC3424190

[B18] Inoue T. Kobayashi Y. Mori N. Sakagawa M. Xiao J. Z. Moritani T. (2018). Effect of combined bifidobacteria supplementation and resistance training on cognitive function, body composition and bowel habits of healthy elderly subjects. Benef. Microbes 9 (6), 843–853. 10.3920/BM2017.0193 30198326

[B19] Jäger R. Zaragoza J. Purpura M. Iametti S. Marengo M. Tinsley G. M. (2020). Probiotic administration increases amino acid absorption from plant protein: a placebo-controlled, randomized, double-blind, multicenter, crossover study. Probiotics Antimicrob. Proteins 12 (4), 1330–1339. 10.1007/s12602-020-09656-5 32358640 PMC7641926

[B20] Karim A. Muhammad T. Shahid Iqbal M. Qaisar R. (2022). A multistrain probiotic improves handgrip strength and functional capacity in patients with COPD: a randomized controlled trial. Arch. Gerontol. Geriatr. 102, 104721. 10.1016/j.archger.2022.104721 35567889

[B21] Kim H. K. Suzuki T. Saito K. Yoshida H. Kobayashi H. Kato H. (2012). Effects of exercise and amino acid supplementation on body composition and physical function in community-dwelling elderly Japanese sarcopenic women: a randomized controlled trial. J. Am. Geriatr. Soc. 60 (1), 16–23. 10.1111/j.1532-5415.2011.03776.x 22142410

[B22] Komaroff A. L. (2017). The microbiome and risk for obesity and diabetes. JAMA 317 (4), 355–356. 10.1001/jama.2016.20099 28006047

[B23] Kondo S. Kamei A. Xiao J. z. Iwatsuki K. Abe K. (2013). Bifidobacterium breve B-3 exerts metabolic syndrome-suppressing effects in the liver of diet-induced obese mice: a DNA microarray analysis. Benef. Microbes 4 (3), 247–251. 10.3920/BM2012.0019 23666099

[B24] Kondo S. Xiao J.-Z. Satoh T. Odamaki T. Takahashi S. Sugahara H. (2010). Antiobesity effects of bifidobacterium breve strain b-3 supplementation in a mouse model with high-fat diet-induced obesity. Biosci. Biotechnol. Biochem. 74 (8), 1656–1661. 10.1271/bbb.100267 20699581

[B25] Lahiri S. Kim H. Garcia-Perez I. Reza M. M. Martin K. A. Kundu P. (2019). The gut microbiota influences skeletal muscle mass and function in mice. Sci. Transl. Med. 11 (502), eaan5662. 10.1126/scitranslmed.aan5662 31341063 PMC7501733

[B26] Lee M. C. Tu Y. T. Lee C. C. Tsai S. C. Hsu H. Y. Tsai T. Y. (2021). Lactobacillus plantarum TWK10 improves muscle mass and functional performance in frail older adults: a randomized, double-blind clinical trial. Microorganisms 9 (7), 1466. 10.3390/microorganisms9071466 34361902 PMC8305125

[B27] Lei M. Hua L. M. Wang D. W. (2016). The effect of probiotic treatment on elderly patients with distal radius fracture: a prospective double-blind, placebo-controlled randomised clinical trial. Benef. Microbes 7 (5), 631–637. 10.3920/BM2016.0067 27633174

[B28] Li Z. Zhang Z. Ren Y. Wang Y. Fang J. Yue H. (2021). Aging and age‐related diseases: from mechanisms to therapeutic strategies. Biogerontology 22 (2), 165–187. 10.1007/s10522-021-09910-5 33502634 PMC7838467

[B29] Liu C. Cheung W. H. Li J. Chow S. K. H. Yu J. Wong S. H. (2021). Understanding the gut microbiota and sarcopenia: a systematic review. J. Cachexia Sarcopenia Muscle 12 (6), 1393–1407. 10.1002/jcsm.12784 34523250 PMC8718038

[B30] Lustgarten M. S. (2019). The role of the gut microbiome on skeletal muscle mass and physical function: 2019 Update. Front. Physiol. 10, 01435. 10.3389/fphys.2019.01435 PMC6933299 31911785

[B31] Nay K. Jollet M. Goustard B. Baati N. Vernus B. Pontones M. (2019). Gut bacteria are critical for optimal muscle function: a potential link with glucose homeostasis. Am. J. Physiol-Endocrinol Metab. 317 (1), E158–E171. 10.1152/ajpendo.00521.2018 31039010

[B32] Ni Y. Yang X. Zheng L. Wang Z. Wu L. Jiang J. (2019). Lactobacillus and Bifidobacterium improves physiological function and cognitive ability in aged mice by the regulation of gut microbiota. Mol. Nutr. Food Res. 63 (22), e1900603. 10.1002/mnfr.201900603 31433910

[B33] Nilsson A. G. Sundh D. Bäckhed F. Lorentzon M. (2018a). Lactobacillus reuteri reduces bone loss in older women with low bone mineral density: a randomized, placebo-controlled, double-blind, clinical trial. J. Intern Med. 284 (3), 307–317. 10.1111/joim.12805 29926979

[B34] Nilsson A. G. Sundh D. Bäckhed F. Lorentzon M. (2018b). Lactobacillus reuteri reduces bone loss in older women with low bone mineral density: a randomized, placebo-controlled, double-blind, clinical trial. J. Intern Med. 284 (3), 307–317. 10.1111/joim.12805 29926979

[B35] Otten B. M. J. Sthijns MMJPE Troost F. J. (2023). A combination of acetate, propionate, and butyrate increases glucose uptake in C2C12 myotubes. Nutrients 15 (4), 946. 10.3390/nu15040946 36839304 PMC9967986

[B36] Petermann-Rocha F. Balntzi V. Gray S. R. Lara J. Ho F. K. Pell J. P. (2022). Global prevalence of sarcopenia and severe sarcopenia: a systematic review and meta-analysis. J. Cachexia Sarcopenia Muscle 13 (1), 86–99. 10.1002/jcsm.12783 34816624 PMC8818604

[B37] Rondanelli M. Gasparri C. Barrile G. C. Battaglia S. Cavioni A. Giusti R. (2022). Effectiveness of a novel food composed of leucine, omega-3 fatty acids and probiotic Lactobacillus paracasei ps23 for the treatment of sarcopenia in elderly subjects: a 2-month randomized double-blind placebo-controlled trial. Nutrients 14 (21), 4566. 10.3390/nu14214566 36364828 PMC9656258

[B38] Rudzki L. Ostrowska L. Pawlak D. Małus A. Pawlak K. Waszkiewicz N. (2019). Probiotic Lactobacillus Plantarum 299v decreases kynurenine concentration and improves cognitive functions in patients with major depression: a double-blind, randomized, placebo controlled study. Psychoneuroendocrinology 100, 213–222. 10.1016/j.psyneuen.2018.10.010 30388595

[B39] Salazar N. Arboleya S. Valdés L. Stanton C. Ross P. Ruiz L. (2014). The human intestinal microbiome at extreme ages of life: dietary intervention as a way to counteract alterations. Front. Genet. 5, 406. 10.3389/fgene.2014.00406 25484891 PMC4240173

[B40] Sandhu K. V. Sherwin E. Schellekens H. Stanton C. Dinan T. G. Cryan J. F. (2017). Feeding the microbiota-gut-brain axis: diet, microbiome, and neuropsychiatry. Transl. Res. 179, 223–244. 10.1016/j.trsl.2016.10.002 27832936

[B41] Sartori R. Romanello V. Sandri M. (2021). Mechanisms of muscle atrophy and hypertrophy: implications in health and disease. Nat. Commun. 12 (1), 330. 10.1038/s41467-020-20123-1 33436614 PMC7803748

[B42] Sharon G. Sampson T. R. Geschwind D. H. Mazmanian S. K. (2016). The central nervous system and the gut microbiome. Cell 167 (4), 915–932. 10.1016/j.cell.2016.10.027 27814521 PMC5127403

[B43] Sugimura Y. Kanda A. Sawada K. Mar Wai K. Asano T. Ozato N. (2022). Association between gut microbiota and body composition in Japanese general population: a focus on gut microbiota and skeletal muscle. Int. J. Environ. Res. Public Health 19 (12), 7464. 10.3390/ijerph19127464 35742712 PMC9224415

[B44] Tosato M. Marzetti E. Cesari M. Savera G. Miller R. R. Bernabei R. (2017). Measurement of muscle mass in sarcopenia: from imaging to biochemical markers. Aging Clin. Exp. Res. 29 (1), 19–27. 10.1007/s40520-016-0717-0 28176249

[B45] van Krimpen S. J. Jansen F. A. C. Ottenheim V. L. Belzer C. van der Ende M. van Norren K. (2021). The effects of pro-pre-and synbiotics on muscle wasting, a systematic review—gut permeability as potential treatment target. Nutrients 13 (4), 1115. 10.3390/nu13041115 33805286 PMC8065581

[B46] World Health Organization [WHO] (2019). World population ageing 2019, 64.

[B47] World Health Organization [WHO]. (2023). Aging and health. [Accessed 2023 September 25]. Available from: https://www.who.int/news-room/fact-sheets/detail/ageing-and-health

