# Supplementary material for: Probiotics supplementation or probiotic-fortified products on sarcopenic indices in older adults: systematic review and meta-analysis from recent randomized controlled trials
Source: Front Aging. 2024 Feb 2;5:1307762. doi: 10.3389/fragi.2024.1307762 (PMC10873954; doi:10.3389/fragi.2024.1307762)
Supplement: Supplementary file 1 [file Table1.DOCX]

| **Supplementary File 1. Search Strategy** | | | | | | |  | | | | |
| --- | --- | --- | --- | --- | --- | --- | --- | --- | --- | --- | --- |
| **Key Concepts** | | | **Concept 1** | **Concept 2** | | | **Concept 3** | | | | |
|  |  |  | **Probiotic** | **Sarcopenia (indices)** | | | **Older Adults** | | | | |
| **Controlled vocabulary terms / Subject terms** | | | “Probiotic” [MeSH Terms] OR “Microbiota” [MeSH Terms] | “Sarcopenia” [Mesh Term] OR “Muscle” [Mesh Term] OR “Muscle Strength” [Mesh Term] | | | “Aged” [MeSH Terms] | | | | |
| **Free text terms / natural language terms** | | | “Probiotic Containing Product” [Text Word] OR “Probiotic Supplementation” [Text Word] | “Muscle Mass” [Text Word] OR “Lean Mass” [Text Word] OR “Total Lean Mass” [Text Word] OR “Muscle Performance” [Text Word] | | | “Older Adults” [Text Word], “Elderly” [Text Word], “Senile” [Text Word] | | | | |
| **Draft Entry EBSCO Search (Identified Articles: 296)** | | | | | | | | | | |  |
| **No.** | | **Entry** | | | **Filter** | | | **Total Findings** | | |  |
| 1 | | (MM "Probiotic*") OR (MM "Microbiota*") OR TX "Probiotic Containing Product" OR TX “Probiotic Supplementation” | | | None | | | 52,906 | | |  |
| 2 | | (MM "Sarcopenia*") OR (MM "Muscle") OR (MM "Muscle Strength") OR TX "Muscle Mass" OR TX "Lean Mass" OR TX “Total Lean Mass” OR TX “Muscle Performance” | | | None | | | 129,382 | | |  |
| 3 | | (MM "Aged") OR TX “Older Adults” OR TX “Elderly” OR TX “Senile” | | | None | | | 1,520,725 | | |  |
| 4 | | S1 AND S2 AND S3 | | | None | | | 296 | | |  |
| **Draft Entry ProQuest Search (Identified Articles: 771)** | | | | | | | | | |  |  |
| **No.** | **Entry** | | | | **Filter** | | | **Total Findings** | | |  |
| 1 | mainsubject(Probiotic*) OR mainsubject(Microbiota*) OR fulltext(“Probiotic Containing Product”) OR fulltext(“Probiotic Supplementation”) | | | | None | | | 71,910 | | |  |
| 2 | mainsubject(Sarcopenia*) OR mainsubject(Muscle) OR fulltext(Muscle strength) OR fulltext(Muscle mass) OR fulltext(lean mass) OR fulltext(total lean mass) OR fulltext(muscle performance) | | | | None | | | 265,261 | | |  |
| 3 | mainsubject(Aged) OR fulltext(older adults) OR fulltext(Elderly) OR fulltext(Senile) | | | | None | | | 3,452,686 | | |  |
| 4 | S1 AND S2 AND S3 | | | | None | | | 771 | | |  |
| **Draft Entry PubMed Search (Identified Articles: 69)** | | | | | | | | | | |  |
| **No.** | **Entry** | | | | | **Filter** | | | **Total Findings** | |  |
| 1 | **"probiotic*"[MeSH Terms] OR "microbiota*"[MeSH Terms] OR "probiotic containing products"[Text Word] OR "probiotic supplementation"[Text Word]** | | | | | None | | | 96,817 | |  |
| 2 | "sarcopenia*"[MeSH Terms] OR "muscles"[MeSH Terms] OR "muscle strength"[MeSH Terms] OR "muscle mass"[Text Word] OR "lean mass"[Text Word] OR "total lean mass"[Text Word] OR "muscle performance"[Text Word] | | | | | None | | | 791,428 | |  |
| 3 | **"aged"[MeSH Terms] OR "older adults"[Text Word] OR "Elderly"[Text Word] OR "Senile"[Text Word]** | | | | | None | | | 3,577,060 | |  |
| 4 | S1 AND S2 AND S3 | | | | | None | | | 69 | |  |
